# Modeling of kyphoplasty cement for accurate dose calculations

**DOI:** 10.1002/acm2.13174

**Published:** 2021-02-18

**Authors:** Per H. Halvorsen, Navneeth Hariharan, Zackary T. Morelli, Ileana N. Iftimia

**Affiliations:** ^1^ Department of Radiation Oncology Lahey Hospital and Medical Center Beth Israel Lahey Health Burlington MA USA

**Keywords:** dose calculation, kyphoplasty cement

## Abstract

We have determined the optimal method for modeling kyphoplasty cement to enable accurate dose calculations in the Eclipse treatment planning system (TPS). The cement studied (Medtronic Kyphon HV‐R®) consists of 30% Barium, 68% polymethylmethacrylate (PMMA), and 2% benzoyl peroxide, formulated to be radiopaque with kV imaging systems. Neither Barium nor PMMA have a high physical density, resulting in different interaction characteristics for megavoltage treatment beams compared to kV imaging systems. This can lead to significant calculation errors if density mapping is performed using a standard CT number to density curve. To properly characterize the cement for dose calculation, we 3D printed a hemi‐cylindrical container to fit adjacent to a micro‐chamber insert for an anthropomorphic phantom, and filled the container with Kyphon cement. We CT scanned the combination, modeled the cement with multiple material assignments in the TPS, designed plans with different field sizes and beam geometry for five photon modes, and measured the doses for all plans. All photon energies show significant error in calculated dose when the cement is modeled based on the CT number. Of the material assignments we evaluated, polytetrafluoroethylene (PTFE) showed the best overall agreement with measurement. Calculated and measured doses agree within 3.5% for a 340‐degree arc technique (which averages transmission and scatter effects) with the Acuros XB algorithm and PTFE as the assigned material. To confirm that PTFE is a reasonable substitute for kyphoplasty cement, we performed measurements in a slab phantom using rectangular inserts of cement and PTFE, showing average agreement of all photon modes within 2%. Based on these findings, we conclude that the PTFE material assignment provides acceptable dose calculation accuracy for the AAA and Acuros XB photon algorithms in the Eclipse TPS. We recommend that the cement be delineated as a structure and assigned the PTFE material for accurate dose calculation.

## 1 INTRODUCTION

Balloon kyphoplasty has become a common treatment for vertebral compression fractures.^1^ A common cement formulation consists of Barium and polymethylmethacrylate (PMMA). Barium ensures that the cement is highly radiopaque when imaged with common diagnostic x‐ray systems. Modern treatment planning dose algorithms rely on voxel‐level corrections for the medium characteristics. Transport‐based algorithms such as Monte Carlo or the Boltzmann‐based Acuros® XB algorithm in the Eclipse treatment planning system (TPS) (Varian Medical Systems, Palo Alto CA) assign a physical material to each voxel and should lead to more accurate dose calculation,^2^ but are more sensitive to mis‐assignment of media.^3^

We have observed very high average x‐ray computed tomography (CT) Hounsfield units (HU), defined as HU = 1000(μ/μ_w_‐1) where μ and μ_w_ are the linear attenuation coefficients in the medium and water, respectively, in CT scans of patients post kyphoplasty. Based on our standard CT scan protocol's 120 kV_p_ energy and associated HU to density conversion table, the material assignment would be in the metal range which is incompatible with the known material composition of the kyphoplasty cement. We therefore endeavored to experimentally determine the optimal material assignment in the Eclipse TPS for a commonly used kyphoplasty cement.

## 2 METHODS

### 2.A Treatment planning system

The Eclipse TPS version 13.7 was used for this work, with both the Anisotropic Analytical Algorithm (“AAA”) and the Acuros External Beam (“Acuros XB”) dose calculation algorithms.^4^ The AAA algorithm uses a 3D pencil beam convolution/superposition method with Monte Carlo‐derived modeling for primary photons, scattered extra‐focal photons, and electrons scattered from the beam limiting devices. A polyenergetic scatter kernel is constructed as a weighted sum of Monte Carlo‐derived monoenergetic scatter kernels, scaled according to the densities of the patient tissue as determined from the HU to electron density table for the relevant CT scan energy. The Acuros XB algorithm uses the linear Boltzmann transport equation (LBTE) to directly account for tissue heterogeneities. The LBTE describes the macroscopic behavior of radiation particles as they interact with matter. In the Acuros XB implementation, the LBTE is solved in an open form using numerical methods by constructing a physical material map based on the physical density inferred from the HU to mass density table or based on manual material assignment, then transporting the photon beam source model into the patient, transporting the scattered photon fluence and electron fluence, and finally calculating the dose (to medium or water). Explicit LBTE solution methods such as Acuros XB are subject to errors from discretization of the solution variables in space, angle, and energy.

### 2.B Anthropomorphic phantom

To enable experimental validation of the kyphoplasty cement with respect to CT scan characteristics, dose calculations, and megavoltage treatment beam interactions, we used the RT‐Safe PseudoPatient^TM^ Prime anthropomorphic phantom (RT‐Safe P.C., Athens Greece) with a custom insert for our A16 micro‐ionization chamber (Standard Imaging, Middleton WI). This configuration provides reasonable density distribution and dimensions,^5^ while ensuring that the medium directly adjacent to the ionization chamber is water, thereby providing a well‐controlled scenario with the introduction of kyphoplasty cement near the ionization chamber. The A16 ion chamber's characteristics^6^ ensure minimal dose gradient across the measurement volume and close proximity to the cement. The high level of “bone” detail in the phantom enables accurate image‐guided localization for high confidence in the measurement location.

The phantom was assembled with the A16 chamber insert and filled with water, and care was taken to purge air bubbles. The phantom was immobilized in the BrainLab radiosurgery mask system (BrainLab North America, Westchester IL), and CT scanning was performed on a Philips Brilliance Big Bore 16‐slice scanner (Philips Healthcare USA, Cambridge MA) using 1.0 mm contiguous slice thickness, 490 mAs, and 120 kV_p_. The image data were imported into the Eclipse TPS and the A16 chamber's active volume and related structures (chamber stem, rod insert) were contoured with high‐resolution settings. For the A16 active volume structure, the structure dimensions were compared to the manufacturer's specifications^6^ to ensure an accurate representation of the collecting volume.

### 2.C Kyphoplasty cement

**FIG. 1:**
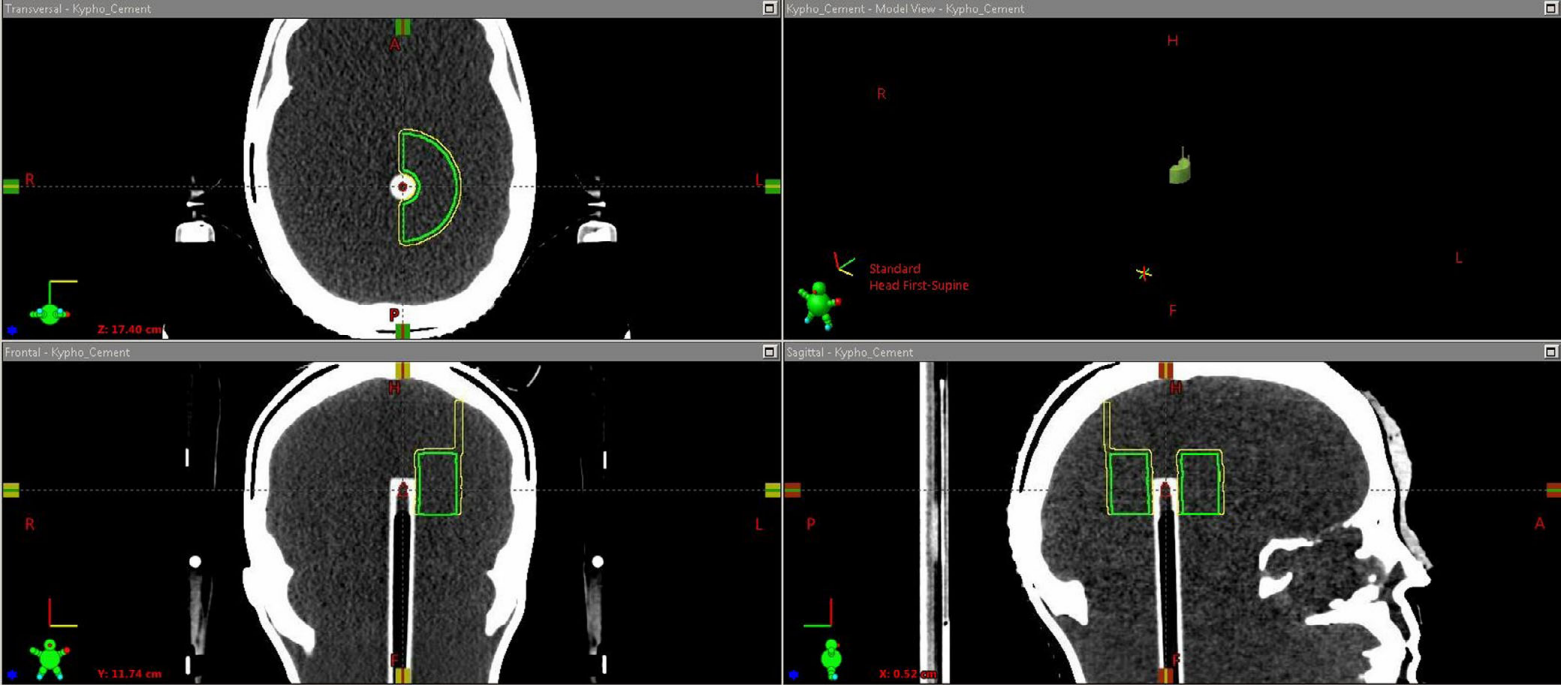
Screenshot from the Eclipse TPS showing the A16 micro‐chamber insert with the active volume contour (red), the planned cement dimensions (green) and the cement container (yellow) with extensions to ensure stable positioning.

A hemi‐cylindrical structure was designed in the TPS with high‐resolution setting, centered cranio‐caudally relative to the A16 active volume structure, with 2.0 cm radial thickness and 3.0 cm cranio‐caudal dimension. In our experience, this represents the maximum clinically realistic dimensions of vertebral kyphoplasty cement. Boolean operations were performed to create a “cement container” structure with 2.0 mm thick walls, modified with two extensions in diagonally opposite corners to ensure reproducible orientation relative to the A16 active volume as shown in Fig. 1.

**FIG. 2:**
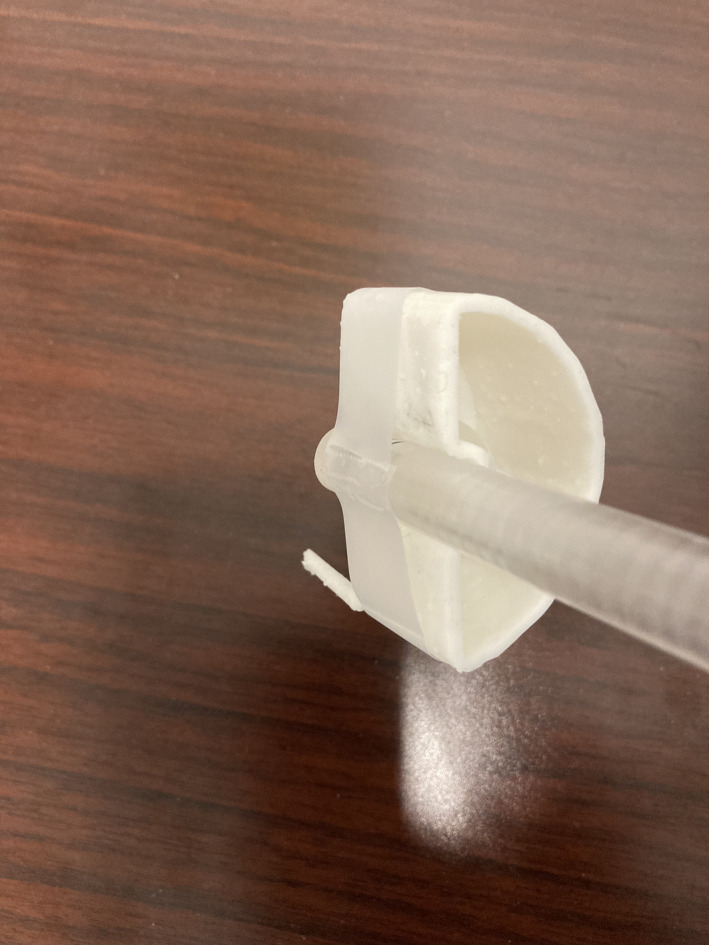
3D‐printed cement container attached to micro‐chamber phantom insert.

The “cement container” structure was exported, processed for 3D printing, and printed using our Form3 printer (FormLabs Inc, Somerville MA), which uses the stereolithography principle^7^ to photopolymerize a resin using a laser capable of 25 micron resolution in order to build 3D objects. The manufacturer's white resin^8^ was used to ensure structural integrity when filled with kyphoplasty cement. The printed object was cleaned, postprocessed and cured following our standard operating procedure. Figure 2 shows the object attached to the phantom's A16 micro‐chamber insert.

**FIG. 3:**
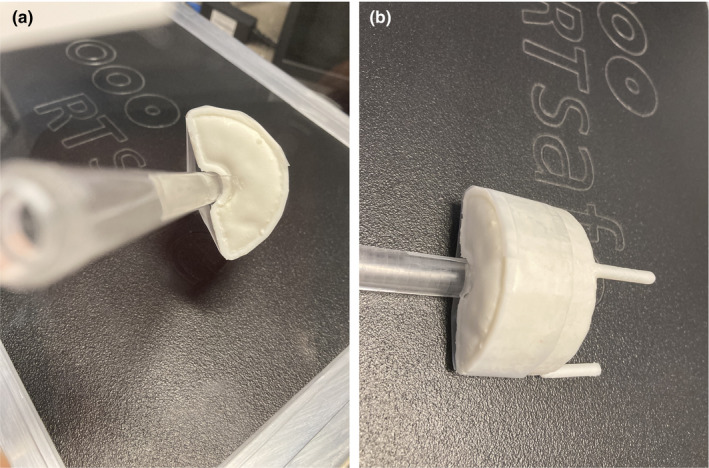
Kyphoplasty cement in 3D‐printed container attached to the phantom chamber insert, (a) on‐end view and (b) lateral view.

The Kyphon HV‐R® kyphoplasty cement (Medtronic Sofamor Danek USA, Memphis TN) was chosen as it is the most commonly used cement in our institution. This cement consists of 30% Barium, 68% PMMA and 2% benzoyl peroxide.^9^ Our institution's Interventional Neuroradiology staff prepared the cement in accordance with their standard operating procedure. When the appropriate consistency was reached, the cement was transferred into the cement holder and allowed to cure, as shown in Fig. 3.

**FIG. 4:**
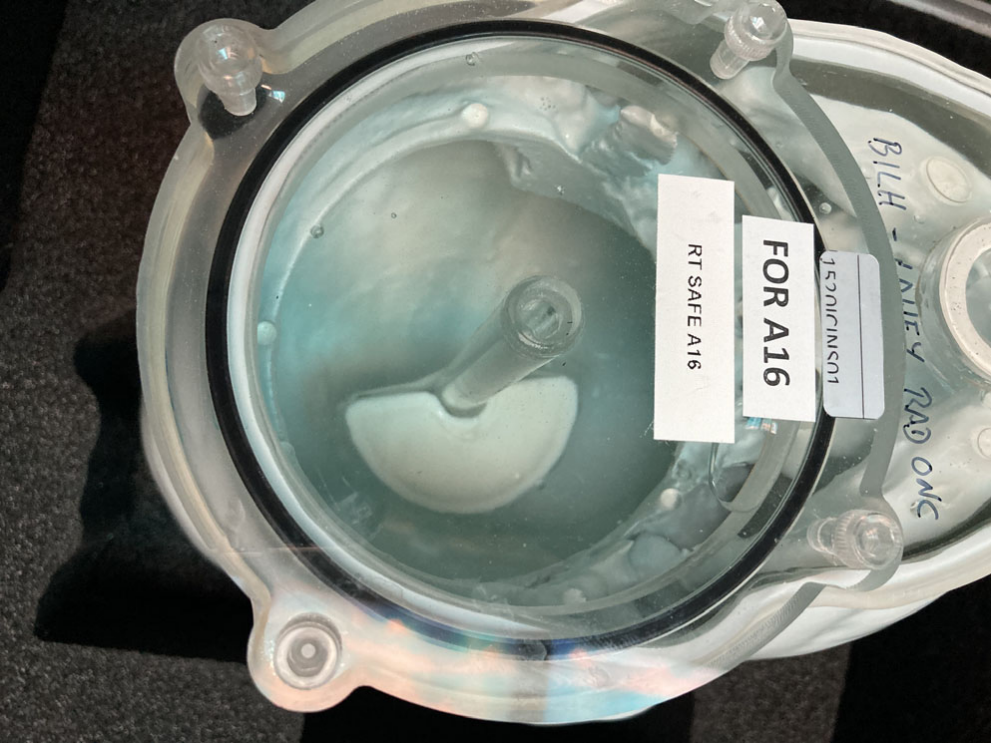
Kyphoplasty cement in anthropomorphic phantom adjacent to ion chamber insert.

The cured cement was mounted directly adjacent to the A16 ion chamber insert, and placed into the phantom which was subsequently filled with water and purged of air bubbles, as shown in Fig. 4. The resulting 7.5 mm distance from the edge of the cement to the A16 ion chamber's collecting volume was chosen to represent the closest distance from vertebral body kyphoplasty cement to the spinal canal, based on our clinical experience, as we believe this represents the most challenging treatment planning scenario involving kyphoplasty cement.

### 2.D Modeling of cement in the TPS

**FIG. 5:**
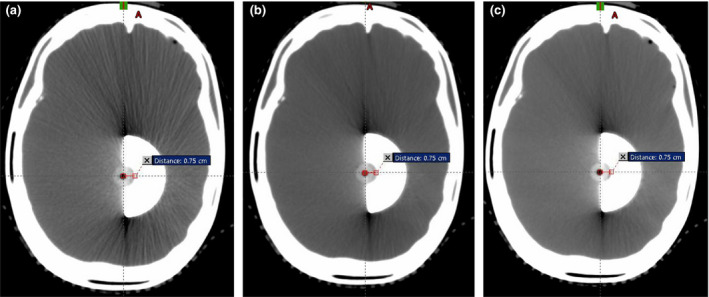
Axial CT slice through the center of the kyphoplasty object, imaged at (a) 90 kV_p_, (b) 120 kV_p_, and (c) 140 kV_p_.

The phantom was immobilized in the BrainLab radiosurgery mask. CT scans were performed with 1.0 mm contiguous slice spacing, maximum mAs for each energy, and energy settings of 90, 120, and 140 kV_p_. The 120 kV_p_ scan was used for dosimetric planning in the Eclipse TPS, as that is our standard energy for treatment planning scans and is the energy used for the HU to density conversion tables in the TPS. The 90 and 140 kV_p_ scans were compared to the 120 kV_p_ scan to assess impact on cement edge delineation and average HU (which would affect density and material assignment if used for dose calculation), as shown in Fig. 5. Our CT scanner uses 12‐bit encoding, with HU = −1024 for 0.0 density and HU = 0 for 1.0 density (mass and electron). Other relevant points include HU = 1200 for 1.69 electron density and 1.56 mass density, and HU = 3071 for 3.79 electron density and 4.59 mass density. The HU artifacts near the corners of the cement insert were overridden for dose calculation.

Multiple copies of the structure set were made in the TPS. For each copy, the density or material assignment of the delineated kyphoplasty cement structure was varied based on the closest matches in elemental composition to the Kyphon HV‐R from the available materials in the Eclipse TPS physical materials library (Acuros XB 13.5 version), and based on the closest assignment based on HU. The resulting assignments were as follows: polyvinyl chloride (PVC), polytetrafluoroethylene (PTFE), titanium (closest physical material based on the average HU, used with the Acuros XB algorithm), and average HU for use with the Analytical Anisotropic Algorithm (AAA). In all structure sets, the A16 active volume was assigned as Water (since the ion chamber correction factor accounts for the air cavity relative to water), and the A16 stem was assigned a CT number of 1600 (based on the chamber's Aluminum electrodes).

A simple beam geometry consisting of two opposed lateral beams was designed in the Eclipse TPS. The beam traversing the kyphoplasty cement prior to reaching the A16 active volume was labeled “Transmission” and the beam entering the A16 active volume from the opposite direction was labeled “Scatter” (due to the close proximity of the kyphoplasty cement immediately downstream from the beam's traverse of the A16 chamber). Three different plans were created, with the following collimator jaw settings: 3 × 3, 5 × 5, and 10 × 10 cm. A 1.0 mm dose calculation grid resolution was used, and each beam was set to 1500 monitor units (MU) for all calculations. Dose was calculated for each field size using both the AAA and Acuros XB algorithms, and for the conventional 6 MV (“6x”) and the 6 MV SRS (“6SRS”) energies on our NovalisTX linear accelerator as well as the 6 MV flattening filter free (“6FFF”), 10 MV (“10x”), and 10 MV flattening filter free (“10FFF”) energies on our TrueBeam STX linear accelerator (Varian Medical Systems, Palo Alto CA). The 6SRS mode uses a smaller flattening filter than the standard 6 MV mode, resulting in a higher nominal dose rate but also a different energy spectrum.

For the 3 × 3 collimator jaw setting, the kyphoplasty cement fully covers the beam aperture. For 5 × 5 cm, the cement covers more than half of the aperture and for 10 × 10 the cement covers less than half of the beam aperture. To illustrate the composite dose effect with modern treatment techniques, a 340‐degree single‐arc VMAT plan was designed using simple optimization criteria (uniform dose across the A16 active volume and dose fall‐off criteria typical for SBRT delivery) without any avoidance zone for the cement. The calculated mean dose to the A16 active volume structure was tabulated for analysis.

### 2.E Dose measurement

Prior to irradiation of the anthropomorphic phantom with kyphoplasty insert, the A16 ion chamber correction factors were determined at 10.0 cm depth for each energy by cross calibration to our ADCL‐calibrated A12 Farmer chamber (Standard Imaging, Middleton WI) using a Solid Water slab phantom (Gammex Sun Nuclear, Melbourne FL). Small‐field correction factor values were determined in accordance with the IAEA code of practice,^10^ using the jaw setting for the collimator‐defined lateral beams and the 50% isodose width for the VMAT plan.

**FIG. 6:**
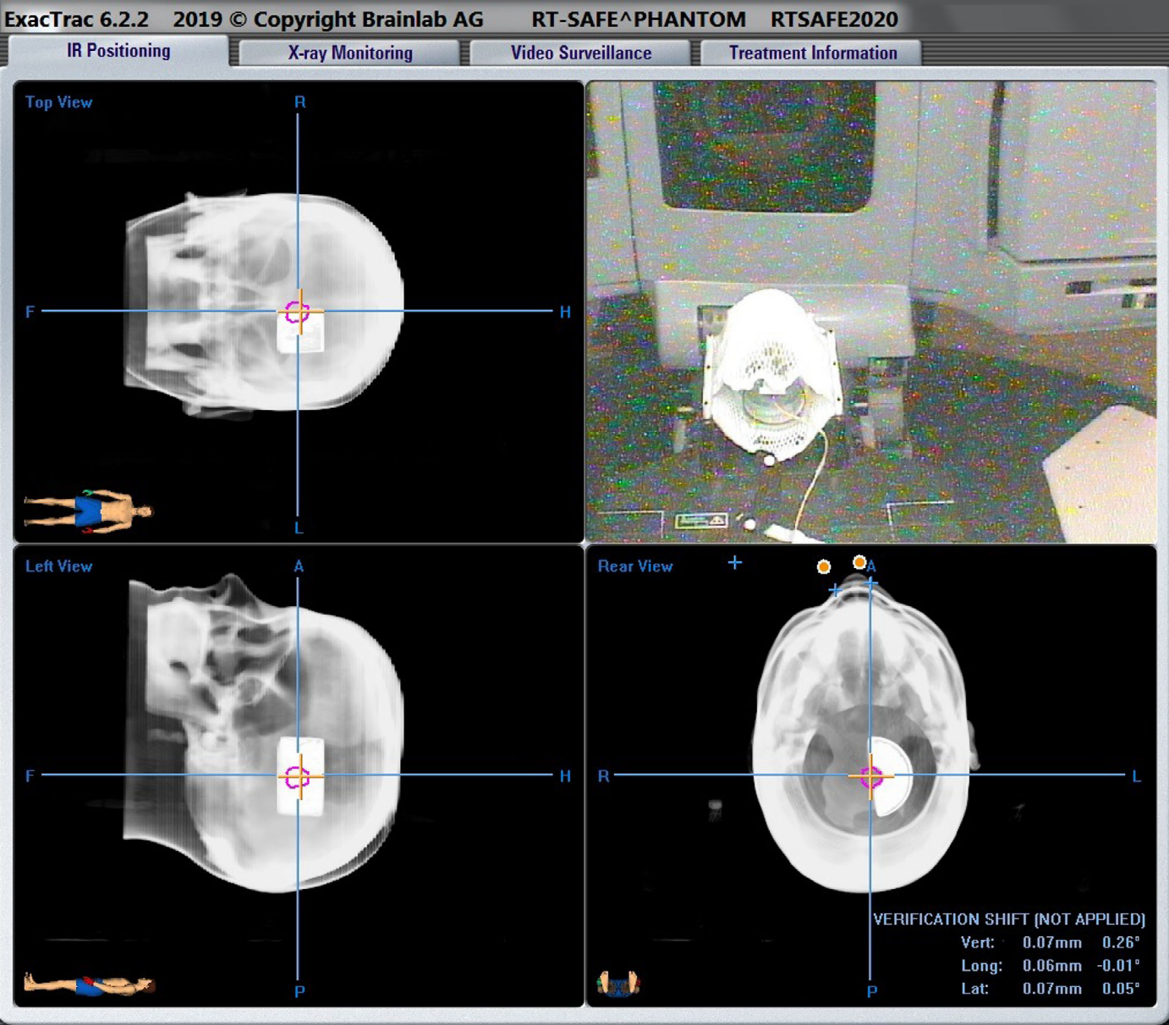
Screenshot showing the use of the ExacTrac system for accurate alignment of the micro‐chamber and kyphoplasty insert for measurement of delivered dose.

One treatment plan per linear accelerator was “Treatment Approved” in the ARIA treatment management system (Varian Medical Systems, Palo Alto CA) and exported to the BrainLab ExacTrac system (BrainLab North America, Westchester IL). This plan was used along with the ExacTrac alignment system to accurately align the phantom and the A16 micro‐chamber on the NovalisTX and TrueBeam STX accelerators, as shown in Fig. 6.

Following confirmation of accurate alignment, the treatment plan arrangements (opposed lateral beams of 3 × 3, 5 × 5, and 10 × 10 field sizes and a 340‐degree arc plan) were irradiated and the dose from each beam was measured for all five photon energies.

### 2.F Slab geometry evaluation

**FIG. 7:**
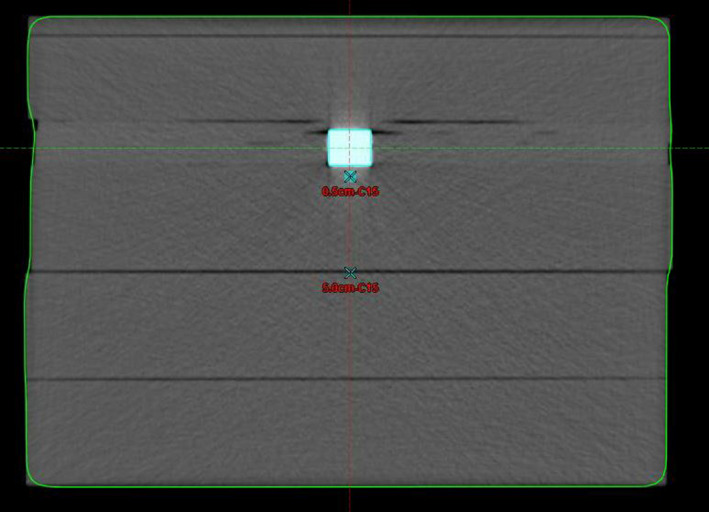
Slab geometry with kyphoplasty cement cut into rectangular shape fitted into custom‐cut sheets of Superflab bolus for measurement at 0.5 and 5.0 cm depths below the cement.

To further explore the agreement between the kyphoplasty cement and PTFE on calculated and measured dose at different distances from these materials, we acquired a solid PTFE rod (United States Plastics, Lima OH). The cured kyphoplasty cement was cut into rectangular pieces 2.0 cm wide and 3.0 cm long, with thicknesses of 1.5 and 0.75 cm, and the PTFE rod was cut into identical dimensions. We used a slab phantom configuration with the isocenter directly behind the insert for simplified geometry, with sheets of Superflab bolus (Eckert & Ziegler, Mount Vernon NY) custom‐cut to hold the inserts with minimal air pockets. An EDGE diode detector (Sun Nuclear Corporation, Melbourne FL) was placed in a custom‐milled Solid Water slab to measure the transmitted dose at 0.5 cm and 5.0 cm depths below the inserts. The detector was previously cross‐calibrated to our ADCL‐calibrated A12 Farmer chamber at 10.0 cm depth. The buildup above the inserts was set to 5.5 cm, and a minimum of 10.0 cm of backscatter material was placed below the detector. Figure 7 shows an axial CT image of the phantom with the 1.5 cm kyphoplasty cement insert. The different thicknesses of kyphoplasty cement also enabled evaluation of CT HU vs volume of cement.

## 3 RESULTS

### 3.A CT image characteristics and TPS contouring

The three CT image sets with different kV_p_ were registered, showing minimal difference in edge delineation with similar window/level settings. However, the mean HU was strongly dependent on kV_p_, as shown in Table 1. As shown in Fig. 5, image artifacts are present near the corners of the cement insert at all kV_p_ scan settings but are more pronounced at 90 kV_p_ (a) and least pronounced at 140 kV_p_ (c) compared to the 120 kV_p_ dataset (b) used for dose calculation.

**TABLE 1:** Mean CT number in the kyphoplasty cement as a function of kV_p_ used for the CT scan acquisition.

kV_p_	Mean CT number in cement (HU)
90	3059.6
120	2479.0
140	2027.4

The mean HU value for the kyphoplasty cement shows a clear dependence on the total volume of cement, as shown in Table 2.

**TABLE 2:** Mean CT number in the kyphoplasty cement as a function of cement thickness.

Thickness (cm)	Mean CT number in cement (HU)
0.75	2940.0
1.5	2690.0
2.0	2479.0

As shown in Fig. 5, the distance from the center of the A16 active volume to the proximal edge of the cement was 7.5 mm.

The “structure statistics” function in version 13.7 of Eclipse reports structure volumes in cm^3^ with one decimal place precision. Consequently, it is not practical to directly compare the manufacturer's stated volume of 0.007 cm^3^ for the A16 ion chamber with the Eclipse calculated volume for the structure representing the A16 ion chamber. The dimensions of the structure representing the A16 ion chamber volume approach the resolution limit of Eclipse contouring, even when contoured as a high resolution structure. The distance from the tip of the structure to the center was 1.6 mm and the axial diameter of the structure was 3.0 mm. These dimensions are within 0.5 mm of the manufacturer's specifications.

### 3.B Anthropomorphic phantom dose measurements

The A16 chamber correction factor values (10 × 10 cm field size, isocentric depth 10 cm) were determined from cross‐calibration to our ADCL‐calibrated A12 Farmer chamber. The resulting correction factors ranged from 370.8 cGy/nC‐MU to 374.6 cGy/nC‐MU. The small‐field correction factors (all energies) were 1.000 for the collimator‐defined fields (3 × 3 and larger), and 1.011 for the VMAT arc based on a 50% isodose width of 1.6 cm.

**FIG. 8:**
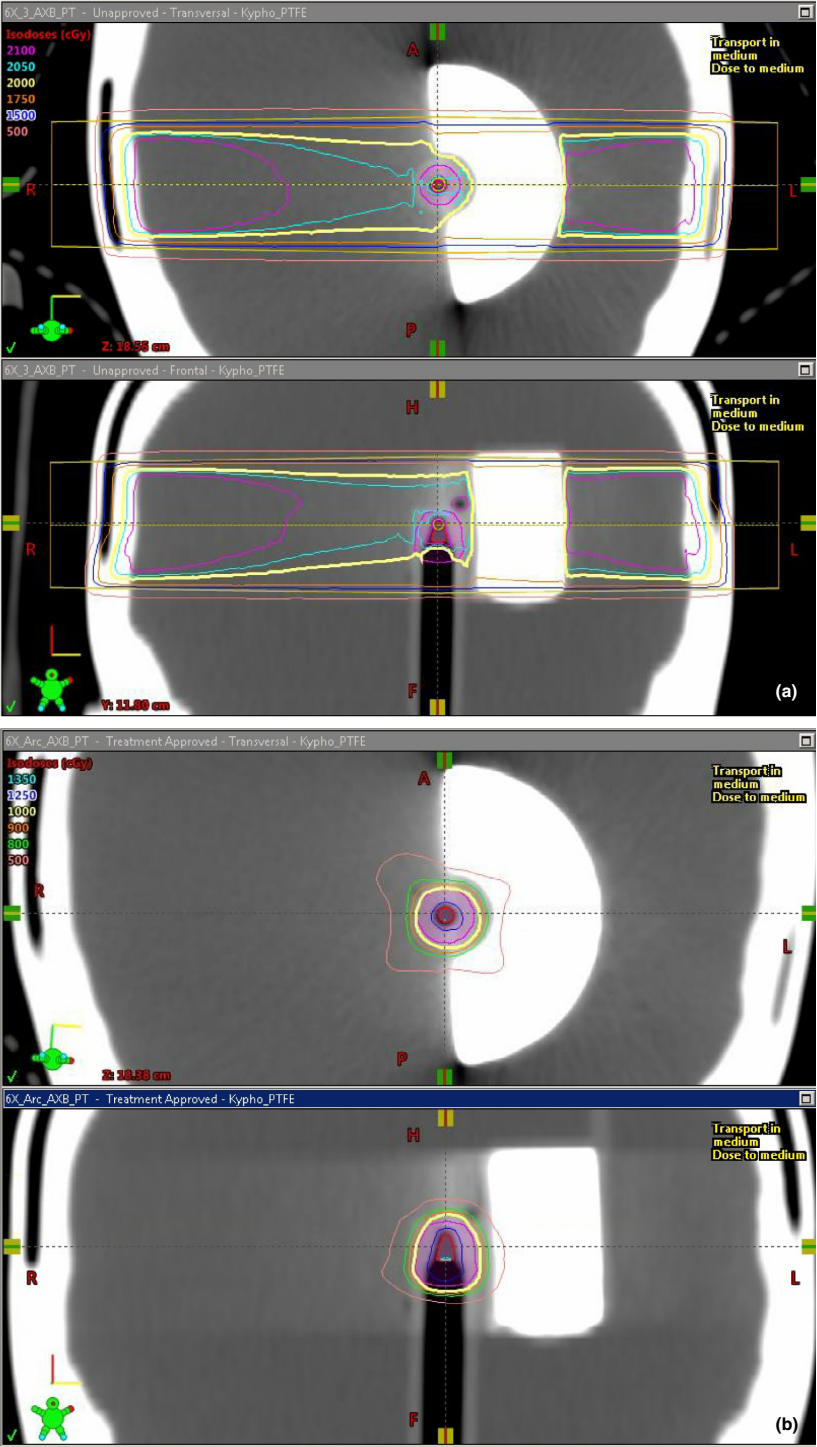
Representative dose distributions for cement modeled as PTFE and dose calculated with the Acuros XB algorithm for the 6MV energy, for (a) opposed lateral 3x3 beams and (b) a 340‐degree VMAT arc.

**FIG. 9:**
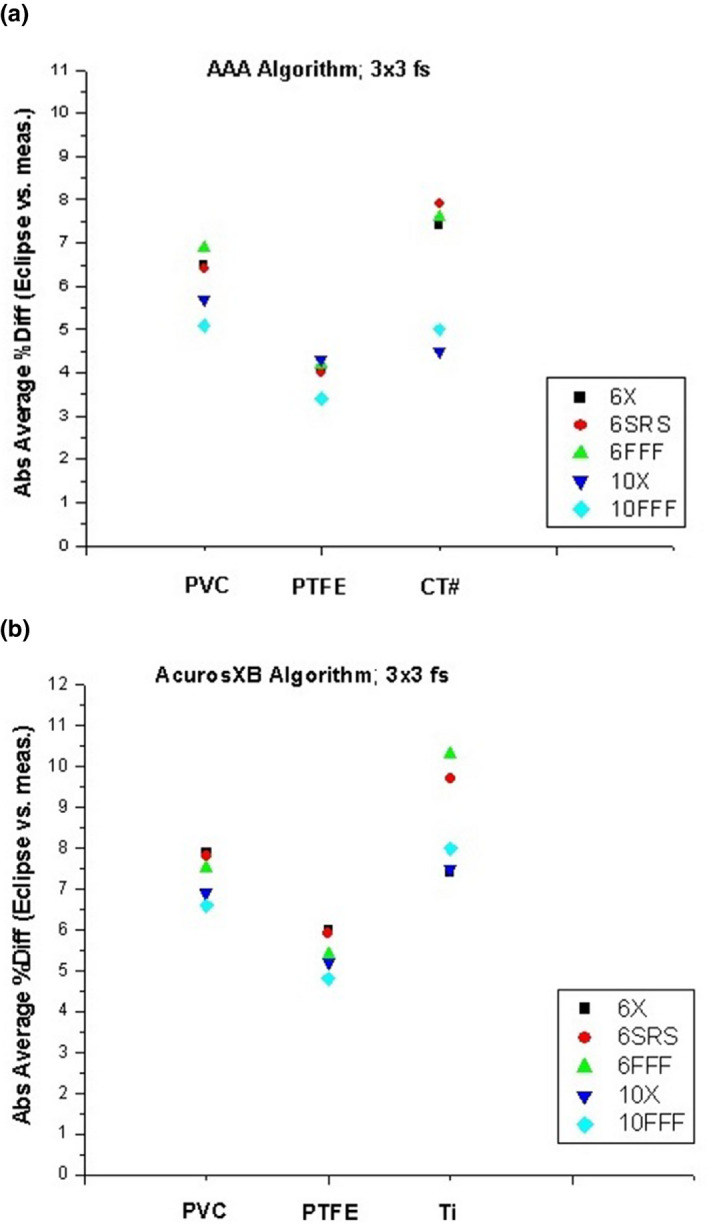
Difference between measured and calculated doses with 3x3 cm field size as a function of photon mode for three different material assignments, for the (a) AAA and (b) Acuros XB algorithms.

**FIG. 10:**
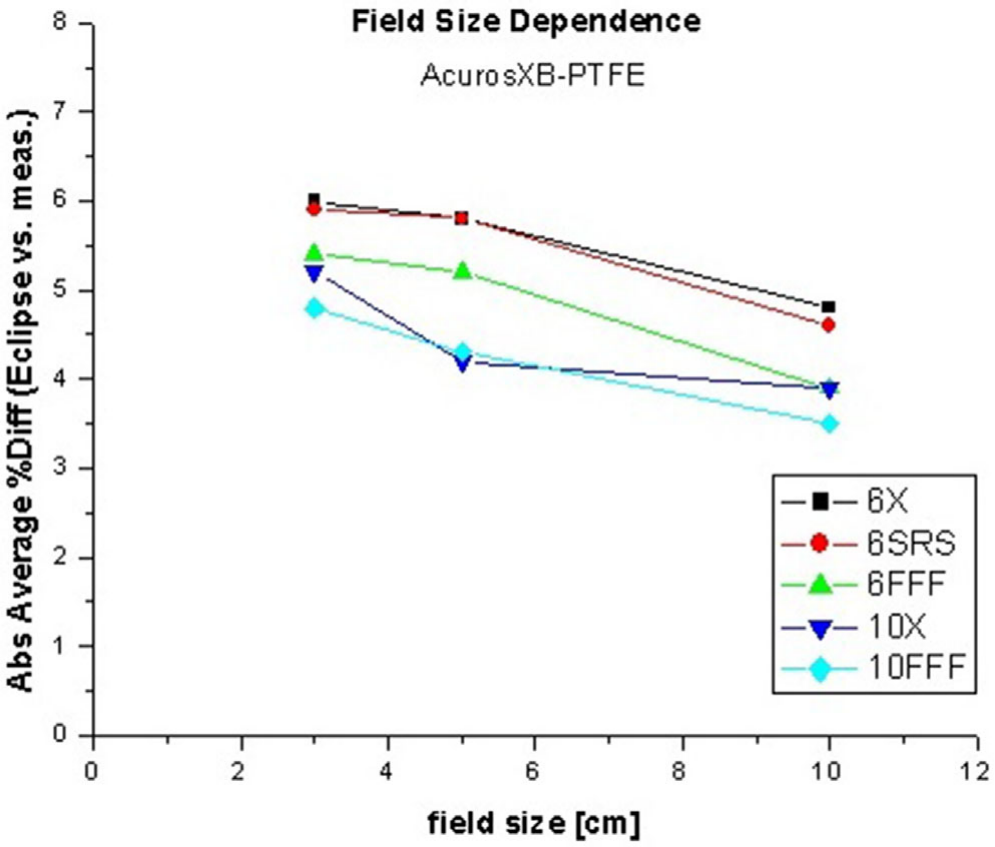
Difference between measured and calculated doses with PTFE material assignment, as a function of field size.

For dose measurements in the anthropomorphic phantom adjacent to the kyphoplasty cement, the aforementioned chamber correction factors and small‐field correction factors based on the IAEA code of practice^10^ were applied in addition to temperature, pressure, and electrometer corrections to determine the measured dose. As previously stated, the Eclipse calculated dose values represent the mean dose to the ion chamber active volume structure in each treatment plan. Representative dose distributions are shown in Fig. 8, and the results are shown in Table 3. The average difference between measured and calculated dose is highest when the CT HU is directly used for dose calculation (either for density scaling with the AAA algorithm or for material assignment with the Acuros XB algorithm). When PVC is assigned to the cement for dose calculation, the average difference is slightly improved but still above 5%. When PTFE is assigned to the cement, the average difference is less than 5% with both algorithms. Figure 9 shows the agreement between measured and calculated doses as a function of photon mode for three different material assignments, for the (a) AAA and (b) Acuros XB algorithms, for 3x3 cm field size. Figure 10 shows the trend with field size.

**TABLE 3:** Calculated doses compared to measured dose for different material assignments, anthropomorphic phantom with largest clinically realistic cement dimensions.

Energy/mode	Field size (cm)	ABS Average %diff (Eclipse calc vs Measured)					
		PVC		PTFE		Titanium	CT HU
		Acuros XB	AAA	Acuros XB	AAA	Acuros XB	AAA
6X	3x3	7.9	6.5	6.0	4.1	9.5	7.4
	5x5	7.5	5.9	5.8	3.9	8.8	6.5
	10x10	6.4	4.8	4.8	2.6	8.6	6.6
	VMAT			3.3	7.0		
6SRS	3x3	7.8	6.4	5.9	4.0	9.7	7.9
	5x5	7.5	5.9	5.8	3.8	9.4	7.4
	10x10	6.2	4.5	4.6	3.3	9.2	7.4
	VMAT			3.3	8.0		
6FFF	3x3	7.5	6.9	5.4	4.2	10.3	7.6
	5x5	7.0	6.6	5.2	4.3	9.8	6.8
	10x10	5.6	5.3	3.9	3.2	9.6	6.9
10X	3x3	6.9	5.7	5.2	4.3	7.5	4.5
	5x5	6.0	4.0	4.2	2.4	7.5	4.4
	10x10	5.7	3.7	3.9	2.2	7.5	4.7
10FFF	3x3	6.6	5.1	4.8	3.4	8.0	5.0
	5x5	6.1	4.2	4.3	2.5	8.1	5.0
	10x10	5.3	3.4	3.5	1.7	8.0	5.2
	VMAT			2.9	6.3		
Average % diff all modes:		6.7	5.2	**4.6**	**4.0**	8.8	6.2

PVC, polyvinyl chloride, PTFE, polytetrafluoroethylene; CT, computed tomography (CT); HU, Hounsfield units.

### 3.C Slab phantom dose measurements

**FIG. 11:**
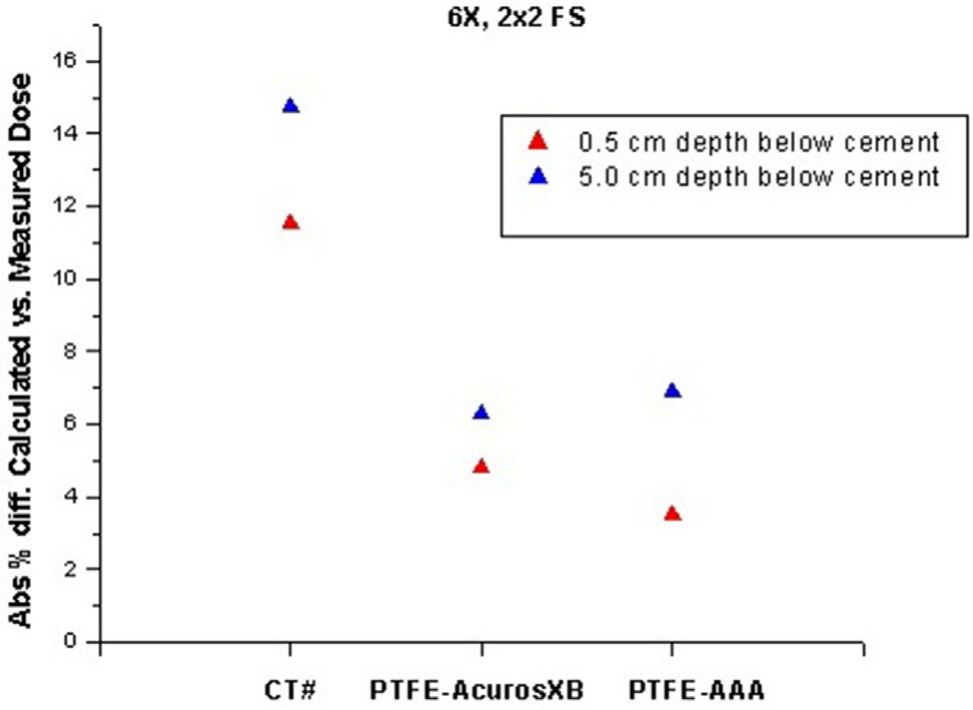
Difference between measured and calculated doses with PTFE material assignment in a slab phantom, at different depths below the kyphoplasty cement.

**FIG. 12:**
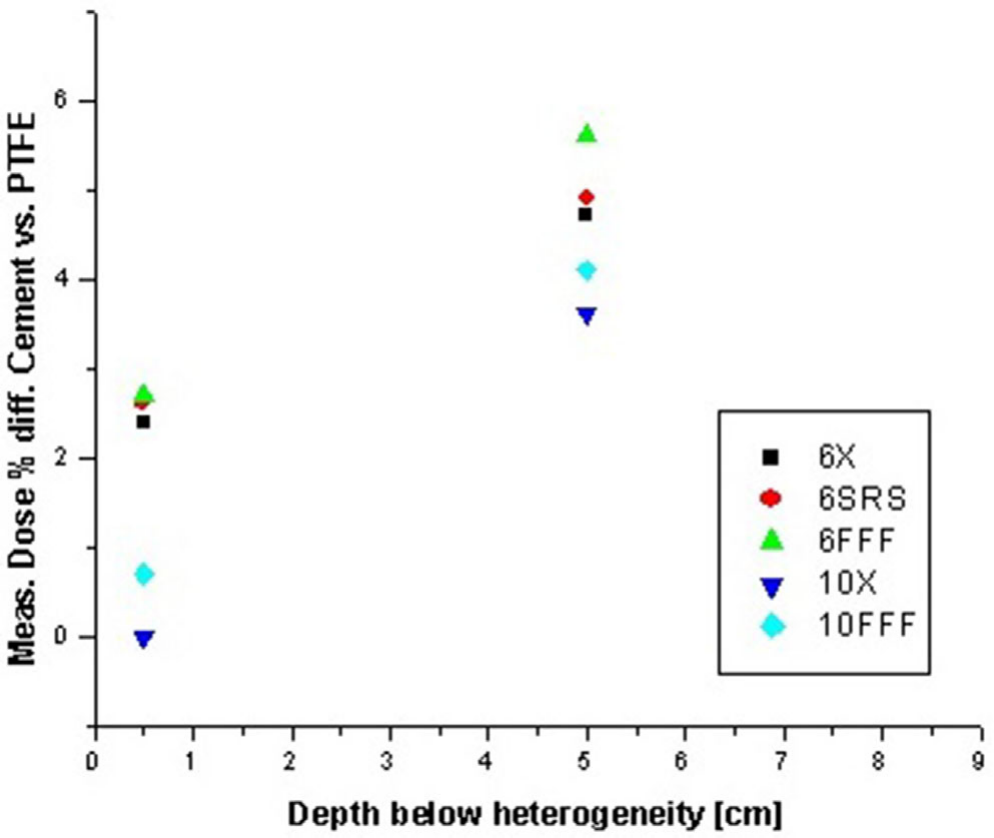
Difference between measured doses at 0.5 and 5.0 cm below kyphoplasty cement and PTFE inserts of the same dimensions.

The EDGE diode detector correction factor values, determined from cross‐calibration to our A12 Farmer chamber at 10 cm depth, ranged from 2.43 cGy/nC‐MU to 2.47 cGy/nC‐MU. Measurements in the slab phantom showed agreement between calculated and measured dose between 0.5% and 2.0% without a cement or PTFE insert, indicating a 2% or better agreement between calculated and measured dose for a simple Solid Water and Superflab slab geometry using 2 × 2 cm collimated fields. Measurements of the same energy/material/depth combinations on different days showed an experimental precision of better than 0.6%. The difference between calculated and measured dose at 0.5 and 5.0 cm below the kyphoplasty cement and PTFE inserts was smaller by a factor of 2 or more for the 0.75 cm thickness compared to the 1.50 cm thickness, as expected. The calculated and measured doses for the 1.5 cm thick inserts are shown in Table 4 and Fig. 11. The average difference across all photon modes at 0.5 cm below the cement insert is 2.9% with the AAA algorithm and 3.2% with the Acuros XB algorithm. Table 5 and Fig. 12 show the difference between measured dose below kyphoplasty cement and PTFE inserts of the same dimensions, thereby excluding any dose calculation variables to directly compare measured dose in the presence of both materials. The average difference in measured dose at 0.5 cm below the inserts across all photon modes is 1.7%.

**TABLE 4:** Calculated doses compared to measured dose for different material assignments, slab geometry with 1.5 cm thick rectangular cement insert and 2 × 2 cm collimated field size.

Energy/mode	Depth below insert (cm)	ABS Average %diff (Eclipse calc vs Measured)		
		PTFE		CT HU
		Acuros XB	AAA	AAA
6X	0.5	4.8	3.5	11.5
	5.0	6.3	6.9	14.7
6SRS	0.5	4.6	3.5	11.8
	5.0	5.7	6.7	14.7
6FFF	0.5	5.1	4.1	13.0
	5.0	6.2	7.4	16.1
10X	0.5	0.0	3.0	2.6
	5.0	2.2	3.1	9.5
10FFF	0.5	1.6	0.3	6.0
	5.0	4.1	4.8	11.8
Average % diff all modes, 0.5 cm depth below insert:		**3.2**	**2.9**	9.0

PTFE, polytetrafluoroethylene; CT, computed tomography (CT); HU, Hounsfield units.

**TABLE 5:** Difference in measured doses with kyphoplasty cement and PTFE inserts.

Energy/mode	Depth below insert (cm)	%diff (measured cement vs PTFE)
6X	0.5	2.4
	5.0	4.7
6SRS	0.5	2.6
	5.0	4.9
6FFF	0.5	2.7
	5.0	5.6
10X	0.5	0.0
	5.0	3.6
10FFF	0.5	0.7
	5.0	4.1
Average % diff all modes, 0.5 cm depth below insert:		**1.7**

PTFE, polytetrafluoroethylene.

## 4 DISCUSSION

We were unable to find peer‐reviewed published articles specifically addressing dose calculation with modern radiation transport algorithms (e.g., Monte Carlo or Boltzmann transport‐based models such as the Acuros XB algorithm) in the presence of kyphoplasty cement. Verhaegen showed significant sensitivity to erroneous physical material assignment in radiation transport dose calculation models.^3^ The AAPM Task Group 105 acknowledged the uncertainty associated with material assignment based on CT number,^11^ and indicated this could lead to significant dose calculation uncertainty with Monte Carlo. Other particle‐transport models such as the Boltzmann‐based Acuros XB model would likely exhibit similar uncertainty. The AAA and Acuros XB algorithms use different methods to align the calculation grid with the image grid, but the differences are negligible for a 1 mm calculation grid resolution combined with a CT image set of 1 mm slice thickness and better than 1 mm in‐plane resolution,^4^ as used in this study. The material assignment method in the Acuros XB algorithm precludes automatic material assignment based on CT HU for volumes larger than an institution‐defined cutoff value; we have set this value to 0.5 cc. Consequently, the kyphoplasty cement and PTFE inserts used in this study had to be manually assigned to a specific material to enable dose calculation with the Acuros XB algorithm, thereby avoiding the potential uncertainty from overlapping density ranges in the automatic material assignment algorithm. Both algorithms use a common dosimetric leaf gap (DLG) value in version 13.7, and this could impact the VMAT plan in this study. The measured DLG value for our TrueBeam STX accelerator's 10FFF mode used in the VMAT plan is 0.36 mm, which agrees well with Kim's published value for the same high‐definition multileaf collimator and photon mode.^12^

A detailed analysis was performed for the experimental uncertainties. The uncertainties^13^ were assessed for each factor used to convert the raw charge reading to dose:

(1) $$Dose=Rdg\times CCF\times SFCF\times P_{T,P}\times P_{\mathrm{el}},$$

where *Rdg* is the raw charge reading, *CCF* is the detector cross‐calibration factor, and *SFCF* is the field size‐dependent small field correction factor. The reading was corrected for temperature, pressure, and electrometer response, since these factors were not included in the CCF.

The planned dose and corresponding MU values were scaled to produce detector raw charge readings in the nC range, and repeated readings showed an uncertainty in the raw charge value of less than 0.5%.

For a given energy, the CCF change for depths and field sizes in the range used in this study is within 0.5%. Based on multiple prior CCF measurements for the same detectors we concluded that the maximum error for this parameter is 1.0%. The SFCF value was based on the IAEA code of practice^10^ using a Boltzmann formula for interpolation. The 50% isodose width was used to obtain the equivalent square field size for the VMAT arc plan. Assuming a 2 mm uncertainty in the equivalent square field size leads to a 0.5% uncertainty in the SFCF value. We estimate the temperature and pressure correction factor uncertainty to be less than 0.3% assuming 1.0°C and 1.0 mmHg uncertainties. The error for the electrometer factor was considered to be negligible.

Detector alignment was performed using the ExacTrac system with a 0.5 mm threshold for robotic couch correction. Assuming 1.0 mm maximum uncertainty in the alignment, we estimate from the VMAT plans that this will result in at most 1.2% change in dose, regardless of the direction of motion.

All these uncertainties were summed in quadrature giving an overall experimental uncertainty for the measured dose of no more than 1.7%.

The kyphoplasty cement used in this study (Medtronic Kyphon HV‐R) consists of 30% Barium, 68% PMMA and 2% benzoyl peroxide. Barium (Z = 56) has a density of 3.6 g/cc. PMMA consists of 60% C, 32% O, and 8% H with a composite density of 1.19 g/cc. The effective density of the cement is therefore approximately 2.05 g/cc with a high‐Z component which provides preferential absorption in the photoelectric range which is predominant with kV CT imaging.

Of the available materials in the Eclipse Acuros XB 13.5 physical materials table, the closest matches are PVC (38% C, 57% Cl, density 1.38 g/cc with no high‐Z component) and PTFE (24%C, 76% F, density 2.20 g/cc with no high‐Z component). For reference, Aluminum (Z = 13, density 2.7 g/cc) and Titanium (Z = 22, density 4.5 g/cc) are also in the materials table.

**FIG. 13:**
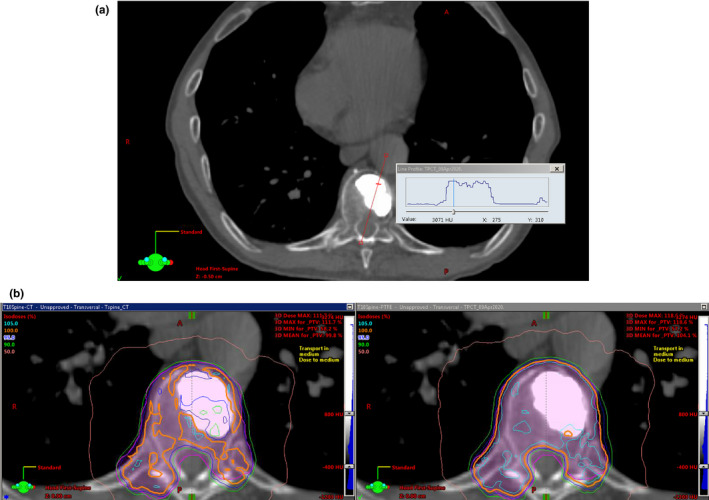
Clinical case involving SBRT to the T10 vertebral body, displayed using a bone window. (a) CT HU profile, and (b) dose distributions showing differences of 5 to 10% in target dose using cement density based on CT HU (left) and PTFE (right).

Using the 120 kV_p_ CT dataset, Titanium would be the closest match in the Eclipse materials table based on the mean HU of 2479. From the results shown in Fig. 7, it is clear that material assignment based on mean HU would result in dose calculation errors near 10% with both algorithms. In the opposed‐field geometry, the underrepresentation of transmitted dose and overrepresentation of backscattered dose may result in acceptable composite dose, but the interface dose would be incorrect and different beam geometries could result in dose calculation errors approaching 10%. Figure 13 shows a typical clinical scenario and the saturated HU value in the cement (a) with the resultant dose difference of 5%–10% in the target (b).

Assigning PTFE as the physical material would result in transmitted dose calculation errors of less than 3.5% and backscatter dose calculation errors somewhat lower than those based on mean HU, and a composite dose calculation error with typical treatment methods in the 3% range based on our 340‐degree arc test. In effect, assigning PTFE as the physical material results in more robust dose calculations that are less sensitive to the beam geometry relative to the cement.

The simplified slab geometry allowed for direct comparison of measured dose with kyphoplasty cement and PTFE, thereby eliminating image artifacts and calculation algorithm considerations. This showed a 1.7% average difference across all photon modes in the measured doses at 0.5 cm below the inserts, and 4.6% average difference at 5.0 cm below the inserts. These results confirm that PTFE is a reasonable material assignment for the kyphoplasty cement used in this study. We consider the average differences between measured and calculated doses (with PTFE as the material assignment) of 3.2% for Acuros XB and 2.9% for AAA at 0.5 cm below the kyphoplasty cement to be quite reasonable given the experimental uncertainty, image artifacts, and calculation algorithm limitations. Clinical applications generally involve the use of multiple beams or arcs, and the impact on total delivered dose would likely be smaller than the aforementioned differences.

The CT scans with three different kV‐range energy spectra clearly illustrate the strong dependence of CT number on the photon energy spectrum and its impact on conversion to density. Other authors have shown that CT scans acquired with MV‐range energy spectra can provide a more accurate representation of the effect on megavoltage treatment beam interactions with dense objects.^14^, ^15^ This is not a practical alternative in most clinics, hence the need for a material assignment methodology based on commonly available kV‐range CT datasets.

## 5 CONCLUSION

Based on direct comparison between calculated and measured doses to a small volume centered at a distance of 0.75 cm from the proximal edge of a 2.0‐cm‐thick hemicylindrical region of kyphoplasty cement and further corroboration in a simplified slab phantom geometry, we conclude that using a material assignment of PTFE provides the most robust agreement between measured and calculated dose for both the AAA and Acuros XB photon dose calculation algorithms in the Eclipse TPS when kyphoplasty cement is in close proximity to an organ at risk.

## CONFLICT OF INTEREST

The authors have no relevant conflict of interest to disclose.
